# Synergistic Antitumor Effect of Doxorubicin and Tacrolimus (FK506) on Hepatocellular Carcinoma Cell Lines

**DOI:** 10.1155/2014/450390

**Published:** 2014-02-20

**Authors:** Francesca Capone, Eliana Guerriero, Angela Sorice, Giovanni Colonna, Gabriella Storti, Jessica Pagliuca, Giuseppe Castello, Susan Costantini

**Affiliations:** ^1^Istituto Nazionale per lo Studio E la Cura dei Tumori “Fondazione Giovanni Pascale”, IRCCS, 80131 Naples, Italy; ^2^Biochemistry, Biophysics and General Pathology Department, Second University of Naples, 80138 Naples, Italy; ^3^Department of Onco-Hematology, S.G. Moscati Hospital, 83100 Avellino, Italy

## Abstract

Hepatocellular carcinoma is the fifth most common cancer worldwide and shows a complex clinical course, poor response to pharmacological treatment, and a severe prognosis. Thus, the aim of this study was to investigate whether tacrolimus (FK506) has synergistic antitumor effects with doxorubicin on two human hepatocellular carcinoma cell lines, Huh7 and HepG2. Cell viability was analyzed by Sulforhodamine B assay and synergic effect was evaluated by the software CalcuSyn. Cell apoptosis was evaluated using Annexin V and Dead Cell assay. Apoptosis-related protein PARP-1 cleaved and autophagy-related protein expressions (Beclin-1 and LC3B) were measured by western blotting analysis. Cytokines concentration in cellular supernatants after treatments was studied by Bio-Plex assay. Interestingly the formulation with doxorubicin and tacrolimus induced higher cytotoxicity level on tumor cells than single treatment. Moreover, our results showed that the mechanisms involved were (i) a strong cell apoptosis induction, (ii) contemporaneous decrease of autophagy activation, understood as prosurvival process, and (iii) downregulation of proinflammatory cytokines. In conclusion, future studies could relate to the doxorubicin/tacrolimus combination effects in mice models bearing HCC in order to see if this formulation could be useful in HCC treatment.

## 1. Introduction

Hepatocellular carcinoma (HCC) is the fifth most common cancer worldwide, and more than half a million new cases occur annually. HCC generally develops from chronic liver injury, which leads to inflammation, hepatocyte regeneration, liver matrix remodeling, fibrosis, and, finally, cirrhosis [1]. The main risk factors for HCC are hepatitis B (HBV) or C virus (HCV) infection, alcohol-induced liver disease (ALD), nonalcoholic fatty liver disease (NAFLD), primary biliary cirrhosis, exposure to environmental carcinogens (particularly aflatoxin), and then type 2 diabetes and obesity [2]. Less common causes include hereditary hemochromatosis, alpha1-antitrypsin deficiency, autoimmune hepatitis, some porphyrias, and Wilson's disease. The distribution of these risk factors among patients with HCC is highly variable, depending on geographic region and race or ethnic group [3]. Although the clinical diagnosis and management of early-stage HCC have improved significantly, its prognosis is still extremely poor [4]. However, this cancer is often diagnosed at an advanced stage when most potentially curative therapies such as resection, transplantation or percutaneous, and transarterial interventions are of limited efficacy and no response is obtained to common therapies. Therefore, new effective and well-tolerated therapy strategies are urgently needed [5, 6]. Chemotherapy is one of the common strategies in HCC treatment, especially for unresectable tumors. Doxorubicin (adriamycin, DOX), antineoplastic chemotherapy drug that is a standard component in treating advanced HCC for its antitumor action, has shown insufficient efficacy, with a response rate of about 15–20% [7]. Other chemotherapeutics, such as epirubicin, cisplatin, 5-fluorouracil, and etoposide and their combinations, demonstrate even lower efficacy [8]. Combined therapy with multiple drugs or modalities is a common practice in the cancer treatment, which can achieve better therapeutic effects than a single drug or modality and can reduce the side effects and resistance to drugs [1]. In fact, it is well known that the cancer is a biological multicellular entity consisting of a heterogeneous population of tumor cells, multiple variety of cell types and normal infiltrating lymphocytes and their communication is bidirectional and dynamic. Therefore, a neoplasm can be defined as a pathological condition resulting from the interaction between tumor and microenvironment in which the latter affects the neoplastic cells growth and their ability to metastasize [9, 10]. Hence, to obtain a new antitumor strategy it is necessary to consider the microenvironment in which the tumor originates and to define the mechanisms that govern the control of immune responses in order to concern the opportunity to use the immunotherapy together with the current cancer therapies. For these reasons, the aim of this study was to combine the doxorubicin with an immunosuppressive agent which could simultaneously both act as an immunosuppressive agent and enhance the chemotherapeutic effectiveness exhibiting antitumor properties. Tacrolimus (FK506) is a macrolide produced by *Streptomyces tsukubaensis* that has shown antiproliferative effects in vitro [11]. Moreover, it has been already used, in patients undergoing orthotopic liver transplantation, as an immunosuppressant able to block the T-cell activation by calcineurin [12]. We have chosen to evaluate the effects of these molecules on two human liver cancer cell lines, Huh7 and HepG2, because our aim is to focus on liver cancer in absence of viral infections. In fact recent papers have evidenced how it is increasing the liver cancer incidence in patients without HBV or HCV infection [13]. This is the reason for which we have already published studies (i) on effects of lipoic and caffeic acids and of sodium selenite on HepG2 and Huh7 cells [14, 15] and (ii) on transcriptomic profile of HepG2 cells compared to normal hepatocytes by microarray experiments [15]. Thereforein this work we have performed the tests also on human cancer cell lines without no evidence of virus genome. Hence, we have investigated the effects of doxorubicin alone and/or combined with tacrolimus on the tumoral cellular growth, cellular death, and tumor microenvironment immunomodulation in order to analyze effect-dose drugs combination on hepatoma cell lines.

## 2. Methods

### 2.1. Cell Culture and Treatments

Human hepatocellular carcinoma cell lines Huh7 and HepG2 (Lonza, Verviers, Belgium) were kept in culture and expanded at 37°C in a humidified atmosphere of 5% CO_2_ in culture medium DMEM (Dulbecco's Modified Eagle's Medium, Lonza), supplemented with FBS (Invitrogen, Camarillo, CA, USA) at 10%, penicillin/streptomycin 100× (Euroclone, Devon, UK), glutamax 100× (Invitrogen), and nonessential amino acids 100× (Invitrogen). Phosphate buffer (PBS phosphate buffered saline Ca^2+^ and Mg^2+^ free) and trypsin (Ca^2+^ and Mg^2+^ free) were supplied by Euroclone. The cells were plated 2 × 10^4^ for well in 96-well tissue culture plates and allowed to attach for 24 h. Experiments were initiated when the cells reached 80% confluence. The cells were treated with doxorubicin (Ebewe Pharma, Unterach am Attersee, Austria) and tacrolimus (FK506) (registered trademark Prograf, Astellas Pharma Inc., TYO: 4503, Japan) for 48 h. The drugs were dissolved in DMEM supplemented with 1% FBS at concentrations of 0.21, 0.42, 0.85, 1.7, 3.4, and 6.8 *μ*M and 0.013, 0.026, 0.052, 0.1, 0.2, and 0.4 *μ*M, respectively, for Huh7 treatment, while the drugs were dissolved in DMEM supplemented with 1% FBS at concentrations of 0.09, 0.18, 0.37, 0.75, 1.5, and 3 *μ*M for doxorubicin and 0.002, 0.006, 0.047, 0.095, 0.18, and 0.36 nM for tacrolimus during HepG2 treatment. Subsequently according to the results, combination treatment (cotreatment) with doxorubicin and tacrolimus was carried out at IC50 (the median inhibitory concentration defined as the drug concentration at which cell growth was inhibited by 50%) doses obtained for 48 h.

### 2.2. Colorimetric Assay with Sulforhodamine B

After 48 h of drugs exposition, cell survival/proliferation was measured in presence and absence of drugs in 96-well plates by a spectrophotometric dye incorporation assay using Sulforhodamine B (SRB). Cells were fixed with trichloroacetic acid (Sigma Aldrich, St. Louis, MO, USA) for 1 h and after stained for 30 min with 0.4% (wt/vol) SRB (Sigma Aldrich) dissolved in 1% acetic acid. The number of viable cells was directly proportional to the protein bound-dye formation which was then solubilized with 10 mM Tris base solution pH 10.5 and measured by fluorometric assay ELISA at 540 nm (Bio-Rad, Hercules, CA, USA; Microplate Reader). All experiments were performed in duplicate and were repeated for three times [16]. The IC50 was assessed from the dose-response curves.

### 2.3. Drug Combination Studies

Drug combination studies were based on concentration-effect curves generated as a plot of the fraction of unaffected (surviving) cells versus the drug concentration after 48 h of treatment. Briefly, individually and in combination equiactive doses (50 : 50 cytotoxic ratio) of the two drugs (IC50) were tested for 48 h. Synergism, additivity, and antagonism were quantified by determining the CI (combination index) calculated by the Chou-Talalay equation and with the software CalcuSyn (Biosoft, Cambridge, UK) [17]. Assuming 0.9 as the cutoff, CI < 0.9, CI(1/4)0.9–1, or CI > 1 indicates synergistic, additive, or antagonistic effects, respectively. The dose reduction index (DRI) represents the measure of how much the dose of each drug in a synergistic combination may be reduced at a given effect level compared with the dose of each drug alone. The linear correlation coefficient (*r*) of the median-effect plot is considered a conformity measure of the data according to the mass-action law principle when the experimental measurement is assumed to be accurate. An *r* value equal to 1 indicates perfect conformity while a poor value may be the result of biological variability or experimental deviations. For all our experiments *r* values were between 0.91 and 0.98 indicating a good data conformity.

### 2.4. Apoptosis Detection

The cells (1 × 10^6^) were harvested and washed twice with ice-cold PBS. Subsequently, the cells were labeled with Annexin V and Dead Cell assay kit according to the manufacturer's instructions (Merck Millipore, Darmstadt, Germany). This assay is based on the phosphatidylserine (PS) detection on the apoptotic cells surface, using fluorescently labeled Annexin V in combination with the dead cell marker, 7-Aminoactinomycin D (7-AAD). We calculated the apoptotic ratio by identification of four populations: (i) viable cells, not undergoing detectable apoptosis: Annexin V (−) and dead cell marker (−), (ii) early apoptotic cells: Annexin V (+) and dead cell marker (−), (iii) late apoptotic cells: Annexin V (+) and dead cell marker (+), and (iv) cells that have died through nonapoptotic pathway: Annexin V (−) and dead cell marker (+). The samples were determined by the Muse Cell Analyzer (Merck Millipore) and analyzed by software provided by Merck Millipore.

### 2.5. Protein Extraction and Western Blotting

Cells were washed once in cold phosphate buffered saline (PBS) and lysed in a lysis buffer containing 50 mM N-(2-hydroxyethyl)-piperazine-N'-2-ethanesulfonic acid, 150 mM NaCl, 1 mM ethylenediamine tetra-acetic acid, 1 mM ethylene glycol tetra-acetic acid, 10% glycerol, 1% Triton-X-100, 1 mm phenylmethylsulfonyl fluoride, 1 lg aprotinin, 0.5 mm sodium orthovanadate, and 20 mm sodium pyrophosphate. The lysates were clarified by centrifugation at 16 000 g for 10 min. Protein concentrations were estimated by a BioRad assay (BioRad) and boiled in Laemmli buffer (Tris-HCl 0.125 m pH 6.8, sodium dodecyl sulphate (SDS) 4%, glycerol 20%, 2-mercaptoethanol 10%, bromophenol blue 0.002%) for 5 min before electrophoresis. Proteins were subjected to SDS-polyacrylamide gel electrophoresis (PAGE) (15% polyacrylamide) under reducing condition. After electrophoresis, proteins were transferred to nitrocellulose membranes (Immobilon-P Millipore Corp., Bedford, MA, USA); complete transfer was assessed using prestained protein standards (BioRad). After blocking with Tris-buffered saline- (TBS-) bovine serum albumin (BSA) (25 mm Tris, pH 7.4, 200 mm NaCl, 5% BSA), the membranes were incubated with the specific primary anti-human antibodies against Beclin-1 (BECN1) 1 : 500 (Cell Signaling Technology, Inc. Beverly, Massachusetts), LC3B 1 : 500 (Cell Signaling Technology), and PARP-1 1 : 500 (Santa Cruz Biotechnology, Inc., Dallas, Texas, USA) overnight at 4°C. When the membranes were washed and incubated with anti-rabbit horseradish peroxidase conjugate at a dilution of 1 : 3000 for 1 h at room temperature, the immunoreactive bands of proteins were visualized by enhanced chemiluminescence immunoassay method (ECL Amersham Biosciences, Little Chalfont, UK). The blots were stripped and reprobed with anti-GAPDH antibody (Santa Cruz Biotechnology) to normalize for differences in protein loading.

### 2.6. Cytokine Evaluations by Bio-Plex Assay

A multiplex biometric ELISA-based immunoassay, the Luminex platform (Bio-Plex, Bio-Rad Lab), containing dyed microspheres conjugated with a monoclonal antibody specific for a target protein was used, according to the manufacturer's instructions (Bio-Plex, Bio-Rad Lab) to evaluate the trends and the concentrations of different soluble molecules in cellular supernatants. After 48 h of incubation with the drugs, used alone or in combination, some cytokines chemokines and growth factors were measured using Human Cytokine 27 Plex Panel (Bio-Plex, Bio-Rad Lab). In particular, we evaluated PDGF-*ββ*, IL-1*β*, IL-1ra, IL-2, IL-4, IL-5, IL-6, IL-7, IL-8, IL-9, IL-10, IL-12, IL-13, IL-15, IL-17, Eotaxin, FGF basic, G-CSF, GM-CSF, INF-*γ*, IP-10, MCP-1, MIP-1*α* (CCL3), MIP-1*β* (CCL4), Rantes, TNF-*α*, and VEGF. Each experiment was performed in duplicate as previously described [16, 18, 19]. Protein concentrations were determined using a Bio-Plex array reader (Luminex, Austin, TX) that quantifies multiplex immunoassays in a 96-well plate with very small fluid volumes. The analyte concentration was calculated using a standard curve, with software provided by the manufacturer (Bio-Plex Manager Software).

### 2.7. Bioinformatics Analysis

The cytokines concentrations evaluated in treated versus untreated Huh7 were compared by *t*-test using the statistical program Prism 4 (GraphPad Software, San Diego, CA, USA). Values of *P* < 0.05 were considered to be statistically significant. Moreover, the possible interactions between the significant proteins were analyzed by Ingenuity Pathway Analysis.

## 3. Results and Discussion

### 3.1. Cytotoxicity Assay

**Figure 1:**
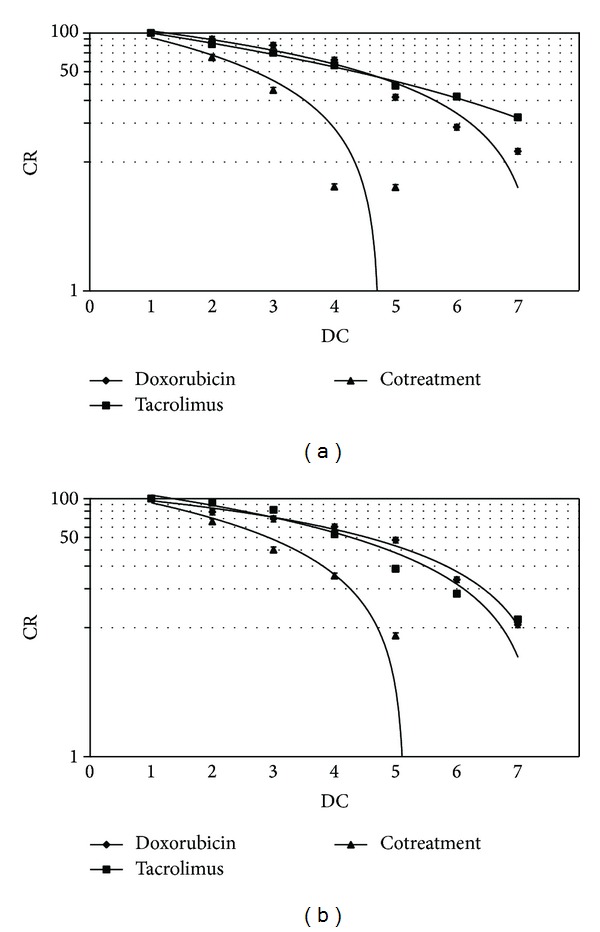
Cell viability %. Huh7 (a) and HepG2 (b) cell lines growth curves (CR) after doxorubicin, tacrolimus, and their combination treatment for 48 h. On the *x*-axis are showed the different drugs concentrations (DC): 1 (no drugs), 2 (dox: 0.21 *μ*M; tacr: 0.013 *μ*M), 3 (dox: 0.42 *μ*M; tacr: 0.026 *μ*M), 4 (dox: 0.85 *μ*M; tacr: 0.052 *μ*M), 5 (dox: 1.7 *μ*M; tacr: 0.1 *μ*M), 6 (dox: 3.4 *μ*M; tacr: 0.2 *μ*M), and 7 (dox: 6.8 *μ*M; tacr: 0.4 *μ*M) for Huh7 and 1 (no drugs) 2 (dox: 0.09 *μ*M; tacr: 0.002 nM), 3 (dox: 0.18 *μ*M; tacr: 0.006 nM), 4 (dox: 0.37 *μ*M; tacr: 0.047 nM), 5 (dox: 0.75 *μ*M; tacr: 0.095 nM), 6 (dox: 1.5 *μ*M; tacr: 0.18 nM), and 7 (dox: 3 *μ*M; tacr: 0.36 nM) for HepG2; on the *y*-axis: cell growth rate (CR).

**Figure 2:**
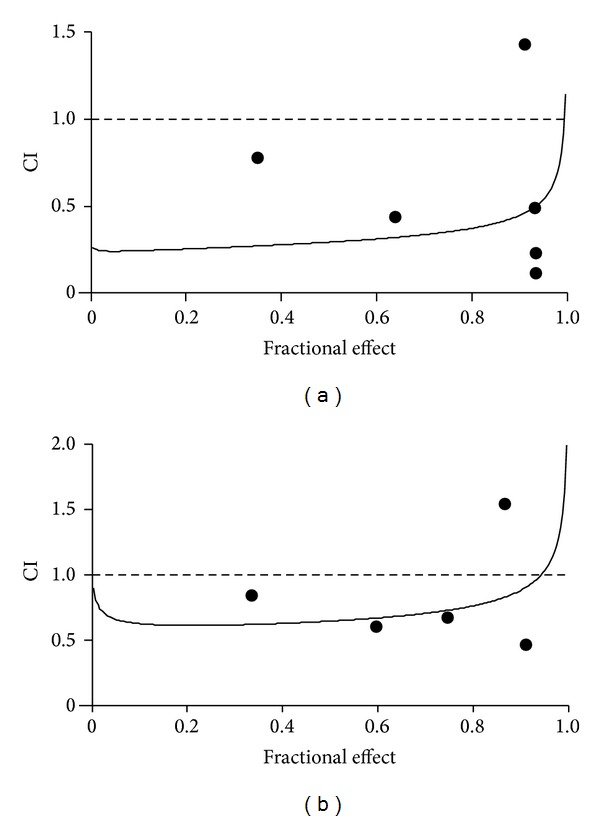
CI/fractional effect curves show CIs versus the fraction of cells affected by simultaneous drugs combination treatment as calculated by median drug effect analysis using CalcuSyn software in Huh7 (a) and HepG2 (b) cells. CI values (black dots) are always below 1 indicating a strong synergistic effect. The points above the CI = 1.0 line represent the maximum drugs concentration used to extrapolate the data using CalcuSyn software.

The cytotoxic effects of doxorubicin and tacrolimus were evaluated on Huh7 and Hepg2 cell lines by SRB assay to identify the concentrations at which cell growth was inhibited by 50%. After 48 h of treatment with doxorubicin and tacrolimus, compared to nontreated cells, Huh7 reached IC50 at 1.1 *μ*M and 0.07 *μ*M doses and HepG2 reached IC50 at 0.72 *μ*M and 0.047 nM doses, respectively (Figures 1(a) and 1(b)). These results indicate that tacrolimus has stronger effect than doxorubicin in inhibiting cell growth of both cell lines tested. By comparison, the IC50 values of both drugs in HepG2 cells were lower than in Huh7 cells. These results suggest that HepG2 cells were more sensitive to these drugs than Huh7 cells. All experiments were performed in triplicate; values were analyzed and presented as means ± standard deviation (SD). Subsequently we evaluated the synergistic antitumor effects of doxorubicin in combination with tacrolimus. Taking advantage of the median drug effect analysis in calculating combination indexes (CIs), we explored the antiproliferative effects of doxorubicin and tacrolimus combinations by testing equipotent doses of the two agents (50 : 50 cytotoxic ratio). A strong synergistic effect with low CIs (CIs < 0.9) was demonstrated when simultaneous equipotent combination doses were used for both cell lines, Huh7 and HepG2 (Figures 2(a) and 2(b); Table 1). In addition, as visible in Table 1, we obtained after the combination treatment a dose reduction in the IC50 values (DRI50) of 5.84-fold for doxorubicin and of 21.15-fold for tacrolimus in Huh7 cells and a DRI50 of 3.35-fold for doxorubicin and of 3.88-fold for tacrolimus in HepG2 cells, compared with the concentrations of the two drugs alone (Table 1).

### 3.2. Apoptosis Studies

Subsequently we investigated the ability of doxorubicin, tacrolimus, and their combination to induce apoptosis in Huh7 and HepG2 cells. Simultaneous treatment with concentrations below the IC50 values of doxorubicin (0.85 *μ*M in Huh7 cells; 0.37 *μ*M in HepG2 cells) and tacrolimus (0.052 *μ*M in Huh7 cells; 0.006 nM in HepG2 cells) induced a synergistic apoptotic effect (Tables 2(a) and 2(b)).

**Figure 3:**
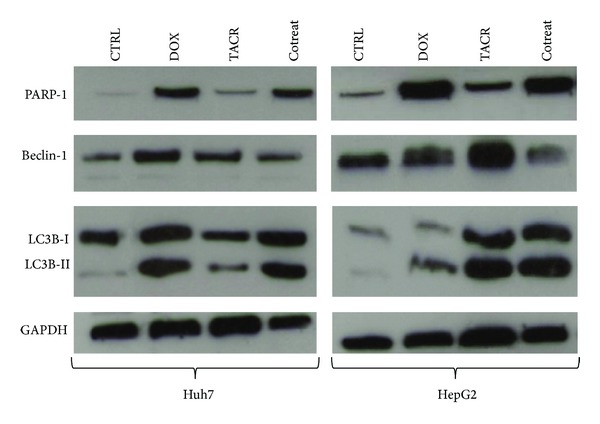
PARP-1 cleaved, Beclin-1, LC3I, and LC3II protein expression analysis by western blotting in Huh7 and HepG2 cell lines after treatment with doxorubicin (0.85 *μ*M) and tacrolimus (0.052 *μ*M) and cotreatment (0.42 *μ*M dox plus 0.026 *μ*M tacr). GAPDH was used as loading control.

In the Tables 2(a) and 2(b) are shown the percentages of the four populations described in Material and Methods Section. These results were further confirmed by western blotting analysis, showing PARP-1 cleavage induction in doxorubicin and combined treatments and only barely detectable correspondent bands after tacrolimus treatment in Huh7 and HepG2 cells (Figure 3).

### 3.3. Autophagy Markers Evaluation

Subsequently we analyzed the autophagy-related hallmarks expression: Beclin-1 (BECN1) and LC3B. Beclin-1 is constitutively expressed in Huh7 and in HepG2 cells (as shown in Figure 3) and participates, together with LC3B-II, in the autophagosome development [20]. In particular, the conversion of LC3B-I to LC3B-II form has been used as indicator of autophagy activation. Based on our result, autophagy may contribute to the survival of HCC during doxorubicin and tacrolimus treatment. In fact cell death was accompanied by a marked proliferation of autophagosomal-lysosomal compartments shown by evident Beclin-1 and LC3B-II bands in both cell lines (Figure 3). On the other hand, during cotreatment there was downregulation of Beclin-1 and upregulation of LC3B-II, being index of autophagy, and we also observed a more enhanced cell death [21, 22].

The mechanism of cell death induced by doxorubicin is discussed in the literature. Doxorubicin induced apoptosis in hepatoma cell lines HepG2 and Bel7402 at concentration of 1.25 mg/L and in Morris Hepatoma cells at concentration of 1 *μ*M [23]. Among the possible cell death mechanisms suggested there is autophagy [8]. Autophagy is an essential and conserved process, through which intact organelles and/or large portions of the cytoplasm are engulfed within double-membraned autophagic vacuoles for degradation, and may be dysregulated in metabolic diseases, neurodegenerative disorders, infectious diseases, and cancer [24]. However, even if basal levels of autophagy ensure the physiological turnover of old and damaged organelles, the massive accumulation of autophagic vacuoles may represent either an alternative pathway of cell death or an ultimate attempt for cells to survive by adapting to stress. In these instances, autophagy operates as a prosurvival mechanism [25]. On the other hand, in the literature it is reported that tacrolimus significantly inhibited the growth of poorly differentiated cell line of HLE and its cytotoxicity was characterized by G0/G1 phase cell cycle arrest [26]. Moreover, it induced cell death of Jurkat human T lymphocytes by apoptosis in dose- and time-dependent manners characterized by nuclear fragmentation and caspase 3 protease activation [12]. It also induced apoptosis by immunosuppression mechanism in T cells [27], produced concentration dependent antiproliferative effect in SMMC-7721 cells (1–100 ng/mL), and its pretreatment for 2 hours enhanced the effect of another drug, the 5-FU, on the induction of apoptosis [11]. Our results showed that during the co-treatment there is a blockage of autophagy. Therefore, the autophagy could be interpreted as a survival mechanism put in place by cells to maintain their function and their viability when they are in conditions of deprivation of energy [21].

### 3.4. Cytokine Evaluations and Interactomic Studies

Then, we evaluated the cytokines levels in Huh7 cellular supernatants after 48 h of incubation with drugs alone and in combination with concentrations below the IC50 values of doxorubicin (0.85 *μ*M) and tacrolimus (0.052 *μ*M) by Bio-Plex assay.

**Figure 4:**
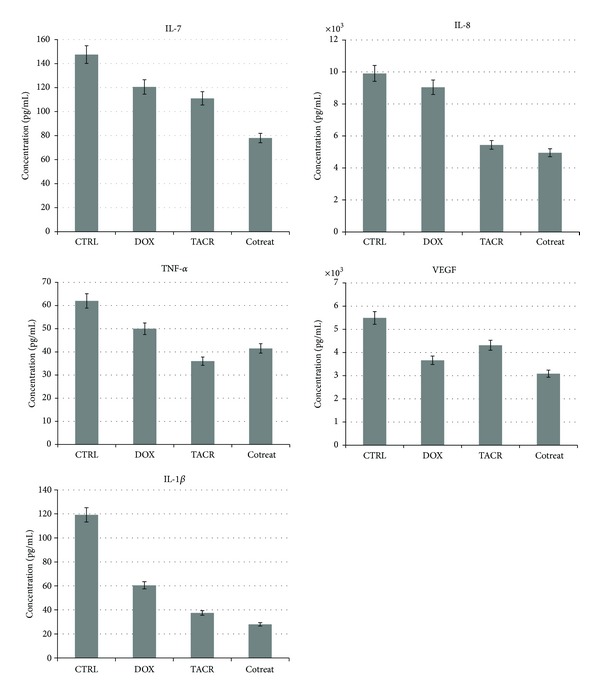
Significant cytokine levels (pg/mL) evaluated in cellular supernatant untreated and after treatment with doxorubicin (0.85 *μ*M) and tacrolimus (0.052 *μ*M) and cotreatment (0.42 *μ*M dox plus 0.026 *μ*M tacr).

The results obtained were compared with untreated cells used as control. These experiments showed that the levels of proinflammatory cytokines, such as IL-1*β*, IL-7, IL-8, VEGF, and TNF-*α* decreased in statistically significant way, with *P*  values < 0.05, both with drug alone rather than cotreatment (Figure 4). Similar results were also obtained for the HepG2 cells (data not shown).

These proteins are strictly involved in HCC. In fact, IL-1*β* is a cytokine that promotes inflammatory processes, stimulating the production of cytokines and the recruitment of other immune system cells and has been found increased in patients with chronic liver disease [28]. IL-8 is a pro-inflammatory chemokine (CXCL8) that has strong pro-angiogenic activity in HCC patients and is upregulated by IL-7 [29, 30]. TNF-*α* plays an important role in apoptosis, in proliferation, in cell differentiation, and in viral replication, and its high expression levels in HCC induce the promotion of cell survival and tumor progression [31, 32]. VEGF is a growth factor upregulated in HCC [33].

**Figure 5:**
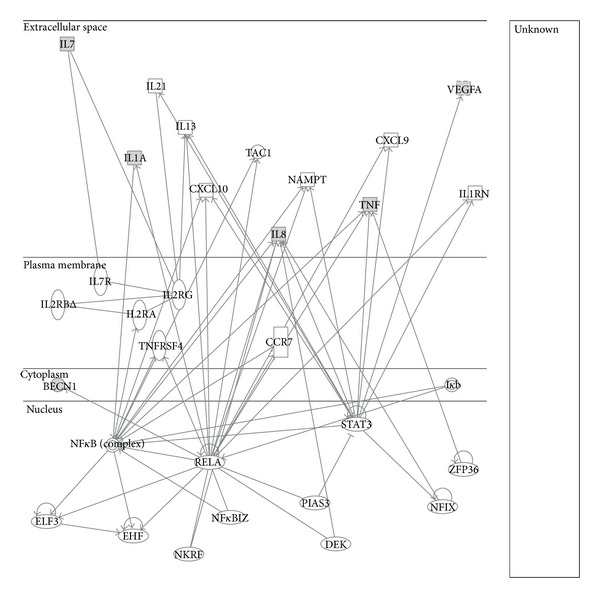
Significant molecules in interactomic analysis by Ingenuity Pathway Analysis (IPA). The interactome shows the close functional association between five significant cytokines and BECN1 (evidenced with cyan symbols) and other functionally relevant molecules (evidenced with white symbols).

Then, the five significant cytokines were analyzed by Ingenuity Pathway Analysis 7.1 (Ingenuity Systems, Inc., Redwood City, CA, USA) that generates networks on the basis of associated functions and data mining from experimental data reported in the literature. Interactomics studies on these cytokines were performed by IPA software. It creates networks between the selected proteins extracting information related to biological correlations from databases that collect experimental data and scientific published papers. The user can insert as input for the research only a list of genes/proteins and not other information like cell types. Our molecules have been found involved into a network named “Cell-To-Cell Signaling and Interaction, Hematological System Development and Function, Immune Cell Trafficking” (Figure 5).

In detail, five significant molecules are connected with some hub proteins, such as NF-*κ*B and its RELA subunit (NF-*κ*B/p65), STAT3 (signal transducer and activator of transcription 3), and ZFP36 (zinc finger protein 36) that are involved in HCC. In fact, in literature it is reported that (i) ZFP36 is responsible for inducing an increase of cell motility and invasiveness in this cancer [31], (ii) STAT3 activation accelerates liver cancer progression [34], and (iii) NF-*κ*B signaling activation induces also HCC metastasis [35]. In detail, in the obtained network, ZFP36 interacts with TNF-*α* interfering with its production by destabilizing its mRNA [36]. STAT3 interacts with IL-8, TNF-*α*, and VEGF by modulating their expression [37]. Finally, NF-*κ*B and its subunit, RELA, are correlated with IL-1, IL-8, and TNF-*α* that interacts with IL-7 creating a heterodimer that contains also their receptors. Moreover, it is very interesting that RELA interacts also with BECN1; in fact, it modulates its transcription and autophagy upregulating BECN1 mRNA and protein levels in different cellular systems [38]. Overall the obtained network shows that five cytokines as well as BECN1 correlated with interesting hub nodes, like NF-*κ*B complex, evidencing that the decrease of their levels can induce NF-*κ*B inactivation able to block HCC development, progression, and metastasis.

In conclusion, our results have shown that doxorubicin and tacrolimus inhibit the growth of both hepatocellular carcinoma cell lines, Huh7 and HepG2, in a dose-dependent manner and their combination induces a more cytotoxic effect. Therefore, we suggest that the mechanisms involved could be a apoptosis induction, contemporaneous decrease of autophagy activation, understood as prosurvival process and immunosurveillance decrement, as well as downregulation of cytokine production. Moreover, it is important to underline that the concentrations used during the cotreatment were lower than IC50 obtained by dose-response assays with each drug treatment. In conclusion, our results indicate that tacrolimus in combination with doxorubicin is expected to be a support drug in HCC treatment. However, these experiments were obtained on cell lines and future studies will regard in vivo experiments to verify if the doxorubicin/tacrolimus combination can have effects in mice models. Until now, there are few studies in this field; in fact, it is resulted in that doxorubicin has improved antitumor activity both in vitro and in vivo with much reduced side effects [39], whereas berberine in combination with doxorubicin suppresses growth of murine melanoma B16F10 xenograft [40].

## Figures and Tables

**Table 1:** Doxorubicin and tacrolimus cotreatment induced a synergistic antiproliferative effect compared to treatment with drugs administered individually as demonstrated by median drug effect analysis calculating the combination index (CI) and the dose redaction index (DRI) with CalcuSyn software. “*r*” is the linear correlation coefficient.

Cell line	Treatment	CI_50_ (±SD)	*r* (±SD)	DRI (dox)	DRI (tacr)
Huh7	dox + tacr	0.22 ± 0.03	0.96 ± 0.01	5.84 ± 0.06	21.15 ± 3.12
HepG2	dox + tacr	0.55 ± 0.03	0.98 ± 0.008	3.35 ± 0.08	3.88 ± 0.05

**Table:** (a)

	CTRL	TACR	DOX	Cotreat
Live	83.34%	31.57%	9.39%	0.81%
Early apoptosis	11.42%	54.18%	1.46%	0.52%
Late apoptosis	4.95%	14.25%	88.96%	98.66%
Dead	0.28%	0%	0.20%	0%

**Table:** (b)

	CTRL	TACR	DOX	Cotreat
Live	70.29%	42.18%	11.51%	3.72%
Early apoptosis	16.70%	28.27%	57.07%	11.62%
Late apoptosis	12.40%	26.96%	30.43%	84.45%
Dead	0.65%	2.61%	1.00%	0.21%
